# Working Women in Large Towns

**Published:** 1890-09-06

**Authors:** 


### Working Women in Large Towns.

### IX.—Domestic Servants.

Hitherto we have been sure of the sympathy of our readers for the working women whose life and labours have been described; we are not quite so sure of it now—as far at least as our lady readers are concerned. Few of us have anything but unconscious relations with the girls who make our matches, our umbrellas, our children's suits, and so forth. But we all have personal relations with members of the class which now come under our notice, and very unpleasant and annoying some of those relations are. Many mistresses think that if a chapter of woes is to be poured forth, the mistress and not the servant should be called upon for it, so stuck up and independent, or else so dirty and incapable, have they found their servants to be. But the very size of the class, if nothing else, makes the omission of it quite impossible in a series of articles upon working women. It has been calculated that there are one million seven hundred and fifty thousand women and girls engaged in domestic service throughout the United Kingdom, and that of these two hundred and twenty-six thousand are to be found in London alone. Besides, while fully admitting that many mistresses are sorely tried, we are sure that servants also have often much to complain of, and that the subject is well worth attention for the sake of both sides.

Unskilled labour can never pay—that is the conclusion to which our investigations in this, as in other branches of our subject, irresistibly impel us. In the tailoring trade, for instance, the employment of unskilled labour gives a tasteless and ill-made coat to the consumer, and to the worker a long day of labour, an empty cupboard, and a bare, cheerless garret. In the servant class, the result is the same, though it shows itself in a different way. The incompetent little "slavey," slurs over her work, breaks the cups and saucers, is dirty and untidy in dress and habits, and becomes the scourge of the family generally; while for her own part her wage is scanty, and her life, unless her mistress is gifted with a preternatural easy-goingness, one long disgrace.

Over eight thousand of these little slaveys come from workhouse schools, being placed out by the guardians at thirteen or fourteen years of age. They have no father or mother, no memories of home; they are perfectly ignorant of the world, with its difficulties and temptations; and the training which they have received, though good in its way, has been a matter of routine rather than of education of the mental faculties. We must choose but one of many stories which show how helpless these girls will often prove to be, when taken from the monotonous round of school life, and set to shift for themselves. The mistress of one of these little girls told her on the first night of her engagement to be sure and put out the candle before she got into bed. More than an hour after, a timid knock came at the door, and a frightened little voice said, "Please, 'm, I've been trying ever since you went down to put this 'ere light out; but I can't find the tap nowhere." Very possibly she had never even seen a candle before; at any rate, another girl, fresh from a workhouse school, had no idea why she was given an umbrella on a wet day, and carried it shut amid a downpour of rain.

These children mostly go into a lodging-house, or take a place in a small family, where only one servant is kept; and a very hard time they often have of it. In lodging-houses the work seems to be never done; and in these, as in private houses, women who take the greatest care of their own daughters often seem to be quite unaware that the slavey is of the same flesh and blood, after all. The baby is teething, and keeps her awake at night, so that she falls asleep while dressing, and this is at once made a ground of complaint against her; while scuttles of coals and cans of water far too heavy for a young girl are handed over to her as a matter of course. The present writer knows of a case in which a bright, healthy little girl (not a workhouse school child) took a place in a dissenting minister's family, and came back after a few months with her health completely wrecked. She was a good, willing child, and carried babies, coals, and pails of water up and down stairs, without a thought of complaining; at last she suddenly collapsed, developed St. Vitus' Dance, was racked with pains, now in one part, now in another, and took many months to recover even a fair amount of health and spirits. She was "overworked," the doctor said; and those who saw the terrible change which those few months of service had wrought were only thankful that she ever recovered at all.

The slavey's life, again, is generally dull as well as hard. Very often she spends the evening all by herself, in an underground kitchen, with not a creature to speak to. Even the cat deserts her for the superior attractions of the parlour rug; and blackbeetles, however much they may endeavour to make up in quantity for what they lack in quality, somehow fail to invite confidences—at least, from the generality of mankind. Her mistress seldom thinks of providing her with books or amusements. Naturally she is woefully dull; and Miss Stanley tells us of one general servant, earning only ten pounds a-year, who was in the habit of spending fourpence a-week upon light and, we fear, not very edifying, literature, so keenly did she feel the need of escaping, if only in spirit, into other scenes, where even murders at least provided some excitement, and gay dresses, jewels, balls, and lovers were everyday affairs. One little servant, the simplicity of whose tale impels us to believe it, is yet worse off in the evenings, being cold as well as lonely. We quote a portion of her letter (given *in extenso* in "Toilers in London"):—

"Dear Commissioner,—Will you tell the servants to ask if a gas-stove is kept before they take a situation, for them stoves is the cruellest things in the winter? My mistress makes me turn the gas off after seven o'clock, and I sit shivering till prayer-time, when I go to bed with cold feet. and I cry sometimes cos I can't well help myself. There is no hot water, becos my mistress has a coal fire in the parlour what she boils a kittle on for the hot water when she goes to bed."

Can mistresses expect to have good and faithful servants when they treat them in this inconsiderate way?

*(To be continued.)*

